# The Phenotype of Mesenchymal Stromal Cell and Articular Chondrocyte Cocultures on Highly Porous Bilayer Poly-L-Lactic Acid Scaffolds Produced by Thermally Induced Phase Separation and Supplemented with Hydroxyapatite

**DOI:** 10.3390/polym16030331

**Published:** 2024-01-25

**Authors:** Wally Ferraro, Aurelio Civilleri, Clemens Gögele, Camilla Carbone, Ilenia Vitrano, Francesco Carfi Pavia, Valerio Brucato, Vincenzo La Carrubba, Christian Werner, Kerstin Schäfer-Eckart, Gundula Schulze-Tanzil

**Affiliations:** 1 Engineering Department, Università degli Studi di Palermo, V.le delle Scienze Building 6, 90128 Palermo, Italy; wallyferraro27@gmail.com (W.F.); aurelio.civilleri@versalis.eni.com (A.C.); camilla.carbone@unipa.it (C.C.); ilenia89v@gmail.com (I.V.); francesco.carfipavia@unipa.it (F.C.P.); valerio.brucato@unipa.it (V.B.); vincenzo.lacarrubba@unipa.it (V.L.C.); 2 Institute of Anatomy and Cell Biology, Paracelsus Medical University, Nuremberg and Salzburg, Prof. Ernst Nathan Str. 1, 90419 Nuremberg, Germany; clemens.goegele@pmu.ac.at (C.G.); c.werner@pmu.ac.at (C.W.); 3 Department of Hematology, Klinikum Nürnberg, 90419 Nürnberg, Germany; kerstin.schaefer-eckart@klinikum-nuernberg.de

**Keywords:** tissue engineering, hydroxyapatite, poly-L-lactic acid, mesenchymal stromal cells, chondrocytes, osteogenesis, chondrogenesis, osteochondral defects

## Abstract

Bilayer scaffolds could provide a suitable topology for osteochondral defect repair mimicking cartilage and subchondral bone architecture. Hence, they could facilitate the chondro- and osteogenic lineage commitment of multipotent mesenchymal stromal cells (MSCs) with hydroxyapatite, the major inorganic component of bone, stimulating osteogenesis. Highly porous poly-L-lactic acid (PLLA) scaffolds with two layers of different pore sizes (100 and 250 µm) and hydroxyapatite (HA) supplementation were established by thermally induced phase separation (TIPS) to study growth and osteogenesis of human (h) MSCs. The topology of the scaffold prepared via TIPS was characterized using scanning electron microscopy (SEM), a microCT scan, pycnometry and gravimetric analysis. HMSCs and porcine articular chondrocytes (pACs) were seeded on the PLLA scaffolds without/with 5% HA for 1 and 7 days, and the cell attachment, survival, morphology, proliferation and gene expression of cartilage- and bone-related markers as well as sulfated glycosaminoglycan (sGAG) synthesis were monitored. All scaffold variants were cytocompatible, and hMSCs survived for the whole culture period. Cross-sections revealed living cells that also colonized inner scaffold areas, producing an extracellular matrix (ECM) containing sGAGs. The gene expression of cartilage and bone markers could be detected. HA represents a cytocompatible supplement in PLLA composite scaffolds intended for osteochondral defects.

## 1. Introduction

Osteochondral defects present an immanent challenge requiring proper cartilage and bone reconstruction [1]. A tissue-engineered construct could help to overcome donor-site morbidity and the limited availability of autografts. To address the microarchitecture of both damaged tissue types, cartilage and bone, a bilayer cell carrier could help [1]. A technique to create layered PLLA scaffold structures was described previously [2]. However, TIPS can also be used to manufacture layered scaffolds [3] since this method allows for obtaining porous scaffolds with interconnected pores, a high degree of porosity and a well-controlled pore size [4].

Hydroxyapatite (HA) is the predominating natural inorganic component of bone and, hence, a promising additive for scaffolds used in bone tissue engineering [5,6]. The osteogenic differentiation of mesenchymal stromal cells (MSCs) could be induced by nano-HA (7%, 0.1–4 µm particle size), which is detectable by gene activity for the early osteogenic transcription factor runt-related protein (RUNX)2, alkaline phosphatase and the main organic component in bone, collagen type 1 [7]. The HA particles were integrated into highly porous composite PLLA scaffolds with pore sizes of 100–150 µm [7]. Nano-HA, in combination with transforming growth factor beta (TGFβ), also stimulated the chondrogenic differentiation of MSCs in PLLA composite scaffolds [8]. PLLA is a versatile, cytocompatible and slowly degradable polymer suitable for the preparation of scaffolds with tailored pore sizes [9]. An increasing HA content impairs the resistance against the deformation of PLLA scaffolds and accelerates scaffold degradation [10]. Since the present study aimed at the future reconstruction of osteochondral defects and not bone defects, a low HA concentration of 5% was selected. A previous study showed osteogenic effects, such as increased alkaline phosphatase gene activity in the dynamic culture of PLLA scaffolds supplemented with 7% HA [7].

Strategies for combining PLLA with HA as a composite were reported previously [7,8,11,12,13]. First in vivo studies of cartilage repair with PLLA scaffolds combined with HA supplementation led to promising results [13,14]. Smaller pores about 100 µm in diameter are more suitable for the maintenance of chondrocyte phenotype, as reported previously [9]. Larger pore sizes are promising for bone tissue engineering with PLLA scaffolds [15]. Taking into account that the ingrowth of vessels during angiogenesis is required for vital bone formation, another study selected pores in the range of 470–590 µm or even larger, as reported in an in vivo study investigating osteogenesis [16]. Accordingly, the adhesion of osteoblasts on PLLA + HA scaffolds produced by thermally induced phase separation (TIPS) was superior on larger pores (500–600 µm) compared to that on smaller pores (150–300 µm) [17]. TIPS is a versatile technique for preparing porous composite scaffolds consisting of PLLA and HA [7,11]. In a previous study, hMSCs were cultured on PLLA scaffolds (mean 100–150 µm pore size) with HA particles under static and dynamic conditions. In contrast to static conditions, dynamic culturing led to superior results in regard to osteogenic differentiation [7]. Another study reported a bilayer scaffold manufactured with different polymer solutions, including chitosan by TIPS, which was tested with cocultures of chondrocyte-like cells (chondrosarcoma cells) and MSCs [18]. The marker expression indicated chondro- and osteogenesis [18]. However, a direct comparison between pure bilayered PLLA scaffolds without and with HA using the TIPS technique as a bilayered osteochondral scaffold carrying cocultures was not performed in previous studies [7].

Hence, the present study aimed to characterize the performance of human bone marrow-derived MSCs alone or in coculture with porcine articular chondrocytes on highly porous bilayer PLLA + HA scaffolds, with a particular focus on their chondro- and osteogenic gene expression.

## 2. Materials and Methods

### 2.1. Materials

Poly-L-lactic-acid (PLLA, Resomer, L 209 S, Evonik Industries, Essen, Germany), 1,4-dioxane (Sigma-Aldrich, Munich, Germany) and commercially available hydroxyapatite HA powder (CamCeram^®^ III, Alhenia AG, Dättwil, Switzerland) were used for scaffold preparation.

### 2.2. Scaffold Preparation

**Figure 1:**
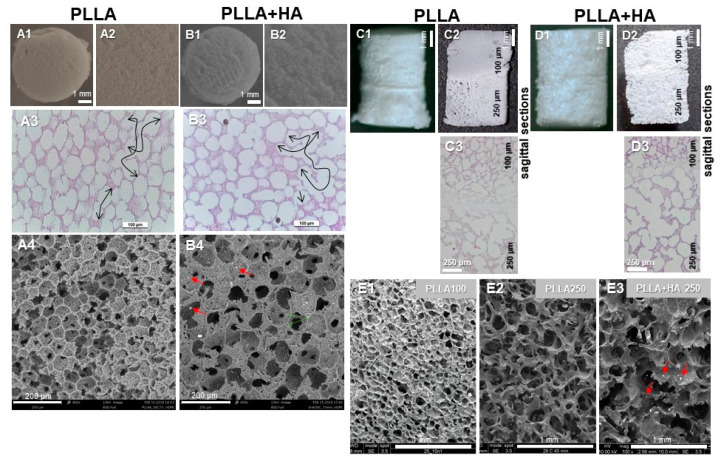
Macroscopical, histological and SEM analyses of PLLA scaffolds without/with HA supplementation. Macroscopical structure of pure PLLA (unilayer: **A1**,**A2**, bilayer: **C1**,**C2**) and PLLA + HA (unilayer: **B1**,**B2**, bilayer: **D1**,**D2**) unseeded scaffolds. Hematoxylin–eosin (HE) staining of unseeded pure PLLA (unilayer: **A3**, bilayer: **C3**) and PLLA + HA (unilayer: **B3**, bilayer: **D3**) scaffolds. (**C3**,**D3**): both layers of bilayered PLLA and PLLA + HA scaffolds. Double-headed arrows (**A3**,**B3**) indicate interconnectivity. Scanning electron microscopy (SEM) of unseeded pure PLLA (unilayer: **A4**, bilayer: **E1**,**E2**) and PLLA + HA (unilayer: **B4**, bilayer: **E3**) scaffold. Scale bars: 1 mm (**A1**,**B1**,**C1**,**D1**), 100 µm (**A3**,**B3**), 250 µm (**C3**,**D3**), 1 mm (**E1**–**E3**), 200 µm (**A4**,**B4**). HA: hydroxyapatite, PLLA: poly-L-lactic acid. Red arrow: HA particles.

Scaffold preparation started with a ternary solution consisting of polymer/solvent/non-solvent. PLLA served as polymer, 1,4-dioxane (Sigma-Aldrich, Munich, Germany) represented the solvent, whereas two times distilled water was the non-solvent. For the unilayer scaffolds (100 µm pore size), the PLLA content was 6 wt%, whereas the ratio of dioxane/water was 87/13 wt/wt. The bilayer scaffolds were produced using 4 wt% PLLA. The weight ratio of the composite scaffold PLLA + HA was 95/5 wt/wt. First, the mixture was treated with ultrasound at 60 °C for 15 min using 35 kHz to evenly disperse the HA particles (0.05–1 µm) with some larger particles (10–100 µm). Subsequently, the solution was filled into cylindrical molds and submerged in a thermal bath with a TIPS time/temperature protocol for PLLA-S (100 µm pore size, S = small) 15 min 25 °C, for PLLA-L (L = large, 250 µm pore size) 45 min 28 °C and for PLLA + HA 45 min at 28 °C (L, 250 µm pore size). Then, controlled cooling was performed by immersing it into an ethyl alcohol reservoir at a temperature of −20 °C for 15 min, followed by freezing the structure. The bilayer structure was obtained by linking two disks of polymer matrices produced with different pore sizes and/or compositions (PLLA-S and PLLA-L without/with HA) by gluing using dioxane. Finally, the foams were gently rinsed using deionized water for removal of any traces of dioxane and vacuum dried for 2–3 h. Pure PLLA scaffolds with comparable porosity and pore sizes obtained at similar conditions were prepared as controls (Figure 1).

### 2.3. Morphological Evaluation of Scaffold Variants

Scaffold topology, pore sizes and particle distribution were observed using a scanning electron microscope (SEM, QUANTA 200F, FEI Company Philips, Thermo Fisher, Waltham, MA, USA) and a micro-computed tomography (CT) scanner (Skyscan 1272, Bruker, Kontich, Belgium).

For SEM observation, the samples were longitudinally fractured using liquid nitrogen to avoid the scaffold’s collapse and were fixed on an aluminum stub. The samples were connected to the sample holding with conductive silver before gold sputtering for 120 s under an argon atmosphere (Sputtering Scancoat Six, Edwards, Crawley, UK), and samples were investigated with an increasing voltage of 10 kV.

The average sizes of pores were calculated by analyzing 8–10 clearly visible pores for each SEM picture by tracing a line between two points (opposite position) of a pore. The morphology and, thus, the size of the HA particles used were studied through an analysis made by MasterSizer2008 (Malvern Panalytical, Malvern, UK). Based on the measurements, the distribution of particle diameters could be extrapolated (Supplementary Figure S1). The final bilayer structure of the scaffolds and the particle distribution were observed via micro CT analysis.

Samples were placed in a scanning chamber. Images were taken at 40 kV, 250 mA, a rotation of 0.1 and pixel size of 4 μm. Reconstructions were performed using the software NRecon (version 1.6.10.2) starting from the acquired projection images. The 2D images had a color depth of 8 bits with 255 grey levels. Finally, the raw image set was 3D visualized by the microCT rendering software CTVox (1.5.2 version).

### 2.4. Pycnometry and Thermal Gravimetric Analysis for Porosity Measurement

Pycnometry was applied to determine overall porosity and interconnectivity of pores in the PLLA scaffolds. For porosity measurements using a gas pycnometer (Pycnomatic ATC, Thermo Fisher Scientific, Waltham, MA, USA), helium was used as a penetrating gas (“helium displacement method”). Using this technique, the material volume of a porous sample was estimated by subtracting the volume of the pores. The shaping of the specimens into a defined cylindrical shape was needed in order to evaluate the bulk volume of the samples. Measurements based on a scaffold mass of about 3.5 mg (neat PLLA scaffolds) and 5 mg (scaffolds supplemented with HA). The actual density of the substance under test is determined by the dry mass-to-volume ratio. Scaffolds were dried before being set into a measuring chamber with a known volume. In a reference chamber that was calibrated, helium was initially charged at a known pressure before being inflated in the room with the sample. Scaffolds’ porosity was calculated using the following expression:

*POROSITY* (%) = 100 × (1 − Vmes/Vapp)

“Vmes” is the real volume of the scaffolds measured by pycnometer, and “Vapp” is the apparent volume calculated with the formula (volume of a cylinder). The amount of HA was calculated by thermal gravimetric analysis (TGA) (Labsys EVO, Setaram Instrumentation, Caluire-et-Cuire, France). Each sample was cut before the measurement chamber was loaded with the sample, which was heated following a temperature profile of 10 °C/minute from 30 °C up to 500 °C under continuous argon gas flow. The analysis of neat PLLA scaffolds served as control.

### 2.5. hMSC Isolation and Culture

hMSCs were derived from bone marrow of ten different healthy donors (mean age = 25.03, 4× female, 6× male). Human bone marrow could be used in accordance with the ethical committee of Bavaria, Germany (Ethical approval number 17,074 from 6 February 2018). In order to separate hMSCs from other cells, the following procedure was followed: bone marrow-derived blood was transferred into T175 culture flasks (6 mL of bone marrow for each flask), each containing 20 mL of hMSC growth medium (Dulbecco’s modified eagle medium (DMEM), PAN-Biotech GmbH, Aidenbach, Germany, enriched with 5% platelet lysate (PL, BioScience, Aachen, Germany), additional supplements were 1% penicillin–streptomycin, 1% amphotericin B, 1% sodium pyruvate (all: PAN-Biotech GmbH) and 0.04% heparin (BioScience)). Bone marrow was cultured at 37 °C and 5% CO_2_. Half of the liquid volume inside the culture flasks was replaced with new growth medium after 92 h to partially remove non-adhering cells different from hMSCs. After 7 days of incubation, the liquid volume was totally changed with fresh culture medium, and when cells were confluent, they were split or cryopreserved. For cell propagation, hMSCs were stripped off with 0.05%/0.02% trypsin/EDTA solution (Merck KGaA, Darmstadt, Germany), rinsed with growth medium and seeded in T175 flasks at 1000 cells/cm^2^. For scaffold seeding, cells were counted with a Neubauer chamber or with a Luna II automated cell counter (Logos Biosystems, Anyang in Gyeonggi-do, Republic of Korea) using 0.4% trypan blue dye in order to exclude dead cells from the count.

### 2.6. Phenotypic Characterization of Undifferentiated hMSCs

The expression profile of cells harvested from human bone marrow was analyzed to identify them as hMSCs by demonstrating typical mesenchymal marker expression by immunolabeling (CD29, CD34, CD44, CD45, CD73, CD90, CD105, vimentin, collagen type 1 and F-actin). For this, hMSCs were seeded at a cell concentration of 9578 cells/cm^2^ on poly-L-lysin (Merck KGaA, Darmstadt, Germany)-coated coverslips and cultured with hMSC growth medium in a 24-well plate at 37 °C and 5% CO_2_ for 7 days. The growth medium was discarded, and the coverslips with hMSCs were rinsed three times with PBS and then fixed with 4% paraformaldehyde (PFA, Morphisto GmbH, Offenbach am Main, Germany) solution for 1 h before being stored in PBS at 4 °C until use for immunofluorescence labeling (see Section 2.10).

### 2.7. Porcine Articular Chondrocyte Isolation and Culture

Articular chondrocytes (pACs) of porcine origin were harvested from non-arthritic knee joints from 4- to 6-month-old female and male porcine donors derived from the slaughterhouse (n = 8). Cartilage slices (1–2 mm) were digested in a rotating bioreactor tube overnight with collagenase NB5 synthesized by *Clostridium histolyticum* (1 mg/mL, Nordmark, Uetersen, Germany) and dissolved in chondrocyte growth medium (96% (*v*/*v*) DMEM/Ham’s F-12 (1:1) with stable L-glutamin, 1% (*v*/*v*) amphotericin B, 1% (*v*/*v*) MEM amino acids, 1% (*v*/*v*) penicillin/streptomycin, 1% (*v*/*v*) ascorbic acid (all: PAN-Biotech GmbH, Aidenbach, Germany). A cell strainer was used (100 µm pore size, TPP, Trasadingen, Switzerland) to remove undigested ECM remnants. Isolated pACs were washed in PBS and cultured in chondrocyte growth medium containing 10% fetal calf serum (FCS) in T175 flasks (CellPlus, Sarstedt AG, Nümbrecht, Germany). At confluence, pACs were stripped off with 0.05%/0.02% trypsin/ethylenediaminetetraacetic acid (EDTA, Carl Roth GmbH and Ko.KG, Karlsruhe, Germany) and propagated in T175 flasks.

### 2.8. Cytotoxicity Testing

For evaluation of cytotoxicity, L929 mouse subcutaneous fibroblasts (strain C3H/An, Cell Line Services, Eppelheim, Germany), which are recommended by the international standard (ISO (International Organization for Standardization) 10993-5:2009 [19]) were cultured at a starting cell number of 1.0 × 10^4^ cells/cm^2^ in flasks at 37 °C and 5% CO_2_ until reaching 80–90% confluence. In addition, hMSCs and pACs were used and seeded in a similar manner. The cytotoxicity testing procedure followed the respective ISO standard. Sterile extracts of all scaffold variants (PLLA without/with HA, 8 bilayer scaffolds per mL respective growth medium) were prepared by incubating them for 48 h in growth medium to determine potential cytotoxic effects. L929 fibroblasts, hMSCs and pACs were seeded at 0.7–1 × 10^4^ cells/cm^2^ in 96-well culture plates for 24 h at 37 °C and 5% CO_2_. Then, 24 h later, the supernatant was completely exchanged by scaffold extracts (volume 100 µL extract/well, triplicates) or control solutions for 24 h at 37 °C and 5% CO_2_. Moreover, 10% dimethyl sulfoxide (DMSO, Carl Roth GmbH and Ko.KG) diluted in the growth medium for each cell type served as a positive control, and growth medium served as negative control. After 24 h of incubation, supernatants were discarded. Instead, 80 µL growth medium and 20 µL [3-(4,5-dimethylthiazol-2-yl)-5-(3-carboxymethoxyphenyl)-2-(4-sulfophenyl)-2H-tetrazolium, inner salt; MTS] solution (CellTiter 96^®^ Aqueous One Solution Cell Proliferation Assay, Promega GmbH, Walldorf, Germany) were filled into each well. Cells were exposed to the reagent for 2 h before absorbance was monitored at a wavelength of 490 nm (Tecan Austria GmbH, Grödig, Austria).

### 2.9. Cell Seeding

Cylindric scaffolds with a diameter of 6 mm and 1.5 mm height (unilayer scaffolds) or 4 mm diameter and 6 mm height (bilayer scaffolds) were colonized with hMSCs alone (unilayer scaffolds) or with hMSCs and pACs (bilayer scaffolds) (see Section 2.9.1 and Section 2.9.2). Each layer was 3 mm high (Figure 1). Scaffolds were disinfected by soaking 70% pure ethanol through them using a vacuum pump (LacaMax GmbH, Essen, Germany) and incubating them overnight in ethanol. Then, disinfected scaffolds were rinsed three times for 20 min in PBS. PBS was soaked through the scaffolds using a steriflip (Merck KGaA). Scaffolds were preconditioned in growth medium for 30 min. Unilayer scaffold cultures were maintained in 96-well plates with 200 µL growth medium, and bilayer scaffold cultures were transferred to a 50 mL TubeSpin bioreactor tube (TPP) after seeding containing 10 mL MSC growth medium placed on an orbital shaker (Bartelt GmbH, Graz, Austria) with 36 rpm rotation.

#### 2.9.1. Unilayer Scaffolds Seeded with hMSCs

At first, unilayer scaffolds were kept immersed in culture medium after the disinfection process; then, they were dried on a sterile gauze for 15 min and positioned in a well of a 96-well plate before being seeded with a cell density of 50,000 cells in 50 µL of cell suspension per scaffold. In each well containing a single scaffold, 50 µL cell suspension was gently poured on the scaffold surface. Thereafter, the well plate was positioned for 1 h inside the incubator for cell attachment onto scaffolds, and, subsequently, additional 150 µL culture medium was added to each well. Growth medium (200 µL) was changed every three days. For osteogenic induction, hMSCs were precultured for 24 h on the scaffolds before MSC medium was exchanged by osteogenic induction medium (DMEM (PAN Biotech GmbH), 10% FBS, 1% penicillin/streptomycin, 2.5% HEPES (Merck KGaA), 0.2% ascorbic acid (Sigma-Aldrich), 1% β-glycerol phosphate and 0.1% dexamethasone (both: VWR, Darmstadt, Germany)).

#### 2.9.2. Flow Seeding Procedure to Coculture hMSCs and pACs on Bilayer Scaffolds

Before starting the seeding, the scaffolds, preconditioned with culture medium, were slightly dried on a sterile gauze to later soak the cell suspension through the structure. The two cell types (hMSCs, passages 2–4, and pACs, passages 2–4) were sown at different time points on each layer in order to limit the mixing of the cells. After detaching the cells, they were suspended in the respective growth media (for hMSCs and for pACs).

The seeding was performed on the same sterile gauze on which the scaffolds were already placed. Due to the flow induced by the water absorbance of the gauze, the cell suspension was soaked into the scaffolds.

The first step was the seeding of hMSCs, whereby a total volume of 810 µL of cell suspension (1000 cells/µL) was gently poured on the layers with larger pore sizes of both scaffold types (without/with HA). More specifically, the cell suspension was poured in steps of 90 µL volume in order to minimize the dispersion of the liquid all over the gauze.

Then, the pACs were added to the scaffold parts with smaller pore sizes with a total volume of 227 µL cell suspension (1000 cells/µL) in 90 µL steps in order to minimize cell loss onto the gauze.

### 2.10. Immunofluorescence Labeling

Coverslips seeded with hMSCs (passages 2–4, 2–7 days of culture) or small scaffold sections (2 mm diameter) were flushed with Tris-buffered saline (TBS: 0.05 M Tris, 0.15 M NaCl, pH 7.6, Carl-Roth GmbH, Karlsruhe, Germany), before being overlaid with blocking buffer (5% donkey serum (Chemicon, Temecula, CA, USA), 0.1% Triton X100 (Sigma-Aldrich) diluted in TBS) for 20 min at room temperature (RT)).

Then, on each sample with fixed cells, 50 µL of a solution containing the respective specific primary antibodies (see Table 1) was poured and diluted in the same blocking solution (dilutions see Table 1) overnight in a humidified chamber at 4 °C. The specimens used as controls were covered with blocking buffer only. The day after, the secondary antibody incubation (see Table 1) and the following staining steps were performed: samples were first rinsed three times with TBS for 5 min each. The secondary antibody solution consisted of cyanine (cy-)3-labeled donkey anti-mouse, donkey-anti-goat or Alexafluor (A) 488-coupled donkey anti-rabbit antibodies with a dilution ratio of 1:200 in blocking solution (see Table 1). 4′,6-diamino-2-phenylindole (DAPI, 10 µg/mL, Roche, Mannheim, Germany) was added to stain the cell nuclei. For CD29 immunolabeling of coverslips seeded with hMSCs, a separated solution was prepared that contained, in addition to the components listed above, Phalloidin A488 (1:100) to visualize the F-actin cell cytoskeleton. The following step was the transfer of the coverslips in a dark and humidified chamber to incubate them for one hour at RT, after which they were washed again three times in TBS, placed on slides and covered with Fluoromount G (Biozol Diagnostica Vertrieb GmbH, Eching, Germany) and an additional coverslip.

### 2.11. Live–Dead Assay

One milliliter of PBS with Ca^++^ and Mg^++^ was mixed with 5 µL of fluorescein diacetate (FDA) stock solution (5 mg/mL in acetone) and 1 µL of propidium iodide (PI) stock solution (2 mg/mL in PBS). The first dye makes living cells green, whereas the second dye stains dead cells red. A total of 50 µL of the staining solution was placed on a thin glass slide, the sample (seeded scaffold segment or coverslip) was immersed, and another 50 µL drop was added to the sample and incubated. After 5 min of incubation, the sample was ready for observation using a Leica Confocal Laser Scanning Microscope (Wetzlar, Germany) with Leica LASX software Version: 3.5.7.23225 to take pictures. Results of the live/dead assay were used to calculate the surface covered by living cells using ImageJ1.48 v software. To assess cell penetration in unilayer scaffolds, cross-sections of the live–dead stained scaffold were performed. In each microscopic field, mean values of minimum of six measurements of the distance between the scaffold surface and the innermost cells visible within the scaffolds were calculated. Bilayer scaffolds were cut longitudinally to check cell immigration into inner parts.

### 2.12. Histological Staining Procedures

For fixation, scaffold cultures were immersed in 4% paraformaldehyde for 24 h (PFA, Santa Cruz Biotechnology Inc.) and stored at 4 °C. Before embedding in paraffin (Merck KGaA), scaffolds were dehydrated in ascending alcohol series (70%, 80%, 96% and 99.6%) followed by xylene. Moreover, 8–10 µm (unilayer or bilayer scaffolds) thick paraffin sections were deparaffinized using 2 × 5 min incubation in xylene and a descending alcohol series. For hematoxylin–eosin (HE) stain, deparaffinized sections were incubated in Harry’s hematoxylin (Sigma-Aldrich) for 6 min, washed in running tap water and immersed in eosin for 4 min (Carl Roth GmbH and Ko.KG). Alcian blue (AB) staining was performed to display content of sulfated glycosaminoglycans (sGAGs). For this, the sections were treated with 1% acetic acid for 3 min, incubated in 1% AB for 30 min (Carl Roth GmbH and Ko.KG), rinsed in 3% acetic acid and washed for 2 min in distilled water (A. dist.). Nuclear fast red aluminum sulfate solution was used for the counterstaining of cell nuclei (Carl Roth GmbH and Ko.KG) for 5 min.

Each staining was covered with Entellan (Merck KGaA).

### 2.13. Trilineage Differentiation of hMSCs Cultured on Coverslips and in Pellet Culture

To prove the multilineage differentiation potential of the bone marrow-derived hMSCs used for the seeding experiments, adipogenic, chondrogenic and osteogenic differentiations were tested. This was performed with hMSCs cultured on coverslips, whereas chondrogenic differentiation was performed with pellet culture. Cell pellets were created by pouring an hMSC suspension containing 200,000 cells into Eppendorf tubes and then by centrifuging them for 5 min at 400 g and depositing them into an anti-adherent 96-well plate after 24 h of incubation in Eppendorf tubes for 4 weeks. Regarding 2D cultures, sterilized poly-L-lysin-coated coverslips were put into a 24-well plate, and cells were seeded at 9579 cells/cm^2^. For adipogenesis, a culture medium with the following composition (volume percentages) was used: DMEM (PAN Biotech GmbH), 10% fetal bovine serum (FBS), 1% penicillin–streptomycin (PAN Biotech GmbH), 0.5% dexamethasone (Sigma-Aldrich), 0.4% insulin (Sigma-Aldrich), 2% indomethacin (Sigma-Aldrich), 0.1% isobutyl-methylxanthine (Sigma-Aldrich), 0.1% rosiglitazone (Cayman chemical company, Ann Arbot, MI, USA), 2.5% 4-(2 hydroxyethyl)piperazine-1-ethanesulfonic acid (HEPES, Merck-Millipore). The composition of osteogenic differentiation medium was already mentioned (Section 2.9.1). Finally, chondrogenic differentiation medium was composed of DMEM (PAN Biotech GmbH), 1% L-glutamine, 2.5% HEPES, 1% penicillin–streptomycin, 1% sodium pyruvate, 0.01% dexamethasone, 0.1% proline, 0.2% ascorbic acid, 1% insulin-transferrin–selenium (Sigma-Aldrich) and 0.01% TGFβ1 (Pepro Tech GmbH, Hamburg, Germany).

### 2.14. Alizarin Red Staining

MSC seeded scaffolds, osteogenically induced MSCs seeded on coverslips and paraffin sections of scaffolds were stained with alizarin red, which forms an orange-red deposit with calcium. Coverslips seeded with osteogenically induced hMSCs were gently rinsed for 5–10 min in A. dist, followed by rinsing three times in TBS at RT, before fixed for 60 min with 60% isopropanol. Then, coverslips were rinsed 2 × 5 min in TBS before being stained with an alizarin red solution (2%, Carl Roth GmbH and Ko.KG) for 5 min. Deparaffinized sections were incubated in alizarin red solution for 2 min and washed with acetone. Then, they were immersed in acetone/xylene (50:50) and, subsequently, in pure xylene before being covered with Entellan.

### 2.15. Oil Red Staining

Oil red staining served to prove adipogenesis with hMSCs. Coverslips seeded with hMSCs were rinsed 5 min with PBS, 5 min with A. dist. at RT, then were fixed for 60 min with 4% PFA before being rinsed again for 2 × 5 min with PBS and for 5 min with distilled water. Cells were fixed and treated in 60% isopropanol (5 min) and then incubated with the oil red staining solution (stock solution 3 g/L: diluted 3:2) for 5 min at RT. A washing step with tap water followed to clear the staining before and after counterstaining with hematoxylin for 30 s. Pictures were taken using a light microscope (DM1000 LED with ICC 50 HD camera, Leica).

### 2.16. Total DNA and GAG Content Measurement

The DNA content of the seeded PLLA and PLLA + HA scaffold variants was quantified using the CyQUANT NF Proliferation Kit (Invitrogen, Eugene, OR, USA) according to the user manual. Samples for the CyQUANT assay were rinsed in PBS (wo Ca^++^, Mg^++^) and immediately frozen, being stored at −80 °C. Unilayer scaffolds were used in total for this assay. In regard to the bilayer assays, both layers were separated using a blade and separately measured. As a standard, calf, thymus DNA (Invitrogen) was applied. All samples were incubated in proteinase K solution (Sigma-Aldrich, 10 mg/mL solved in 50 mM Tris/HCl, 1 mM EDTA, 0.5% Tween20, pH 8.5) for 16 h at 55 °C. Then, specimens were centrifuged at 10,000× *g* for 30 min, and the supernatants were stored at 4 °C. Based on the information that each cell possesses, in mean, 7.7 pg DNA, as reported previously for chondrocytes [20], the DNA content of each specimen was calculated. sGAG contents were measured using the dimethylmethylene blue (DMMB) assay. The specimens were diluted in phosphate-buffered EDTA buffer (PBE, 100 mM Na_2_HPO_4_, and 5 mM EDTA, pH 8) before the addition of the DMMB staining solution (8.9 mM DMMB hydrochloride in 600 mg glycine, 467 mg NaCl and 200 mL A. dist.). Immediately thereafter, the absorption shift from λ = 525 nm to λ = 595 nm was assessed (Tecan reader, Groedig, Austria). sGAG content was calculated with chondroitin sulfate (Sigma-Aldrich) as a reference standard.

### 2.17. RNA Isolation

Colonized bilayer scaffolds were snap-frozen at −80 °C. The samples were incubated with 1000 µL of pre-cooled Qiazol (Qiagen, Hilden, Germany) and disintegrated using a tissue lyser (Qiagen) at 50 Hz for 2 × 5 min. After incubating for 5 min at RT, 200 µL of 1-bromo-3 chloropropane (Sigma-Aldrich) was added. Centrifugation of the specimens for 15 min at 4 °C with 12,000× *g* followed, with the aim of obtaining a pure supernatant containing RNA which was pipetted on Qiashredder columns (Qiagen). Isolation of RNA followed the RNeasy Mini kit protocol (Qiagen) with an on-column DNase digestion. The Nanodrop ND-1000 spectrophotometer (Peqlab, Biotechnologie GmbH, Erlangen, Germany) was used to determine quantity and purity (260/280 absorbance).

### 2.18. Gene Expression Analysis of Bilayer Scaffolds Seeded with pACs and hMSCs

For each sample, 600 ng of total RNA was reversely transcribed into cDNA using the reverse transcription kit (QuantiTect Reverse Transcription Kit, Qiagen) according to the manufacturer’s protocol. Quantitative real-time PCR (qRT-PCR) reactions were based on 20 ng cDNA and the TaqMan Gene Expression Assay (Life Technologie, Darmstadt, Germany) with primer pairs for *collagen type 2 (COL2A1)*, *collagen type 10 (COL10A1)* and *runt-related protein (RUNX) 2* and the reference gene *beta actin (BAC)* for hMSCs and pACs (Table 2). For qRT-PCR, the StepOnePlus (Applied Bioscience [ABI], Foster City, CA, USA) thermocycler with the program StepOnePlus software 2.3 (ABI, Foster City, CA, USA) was applied. Normalization to the reference gene (*BAC*) was performed before calculating the relative gene expression of each analyzed gene. For calculation, the ΔCT method, as described [21], was applied.

### 2.19. Statistics

Mean values and standard deviation are shown for each data. Statistic analysis was performed using GraphPad Prism5 (version 5.02, GraphPad Software, Boston, MA, USA). By means of Rout test, outliers could be identified if n ≥ 5. In the case of comparing one group (e.g., PCR data) with respective controls, the one-sample *t*-test (two-tailed) was applied. The comparison of two groups with each other was performed with One-way ANOVA (CyQuant, DMMB assays) in combination with Tukey’s multiple comparison test. Statistical significance was stated at a *p*-value of ≤0.05.

## 3. Results

### 3.1. Structure of Unseeded Scaffold Variants as Determined by SEM, Pycnometry and Histology

All scaffold variants had a highly porous structure with interconnecting pores (Figure 1, arrows). The unilayer scaffolds without/with HA had a mean pore size of 100 µm as calculated by SEM. A mean porosity of 90 ± 2% was determined with pycnometry and water displacement for the unilayer scaffold variants. Both layers of the bilayer scaffolds showed >93% overall porosity, measured by pycnometry, and a mean pore size of 100 µm in the upper layer of neat PLLA mimicking the cartilage layer and of 250 µm in the bottom layer simulating bone consisting of either PLLA + HA or neat PLLA as a control. The walls of the pores in all scaffold variants contained small micropores visible in the HE staining and SEM. Particles of HA (Figure 1, red arrows) were evenly distributed and visible in SEM. Scaffold structure and surface seemed not to be affected by the addition of HA (Figure 1).

**Figure 2:**
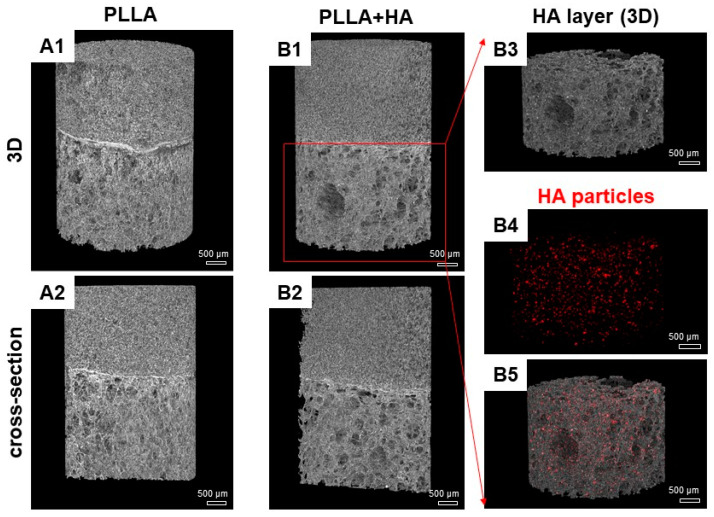
(**A**,**B**): Representative microCT images of bilayer scaffolds. Three-dimensional view on pure PLLA and PLLA + HA scaffolds (**A1**,**B1**) and cross-sectional views (**A2**,**B2**). MicroCT image (3D) of the isolated HA scaffold segment (**B3**), visualization of the HA particles in the HA segment of the bilayer scaffold (**B4**, red), merged view of **B3** + **B4** for localization of the HA particles in the HA layer (**B5**). HA: hydroxyapatite, PLLA: poly-L-lactic acid. Scale bars: 500 µm.

MicroCT analysis was undertaken to obtain a deeper insight into scaffold microstructure, the linkage between both layers (Figure 2(A1,A2,B1,B2)) and HA particle distribution (Figure 2(B3–B5)). The results were compared with those of SEM. For both scaffold variants (pure PLLA and PLLA + HA), it could be observed that the linking process between the two sub-units was successfully carried out. As a matter of fact, the subunits adhered well to each other, thus generating a continuous scaffold matrix. Images confirmed the uniform distribution of small HA particles embedded in the polymer matrix or attached to the pore walls (Figure 2(B3–B5)). Most of the HA particles had a size of (0.05–1 µm) with a few larger particles (10–100 µm), as revealed by particle size analysis (Supplementary Figure S1A). This result was confirmed by SEM analysis (Supplementary Figure S1B).

### 3.2. Cytocompatibility of the Scaffold

**Figure 3:**
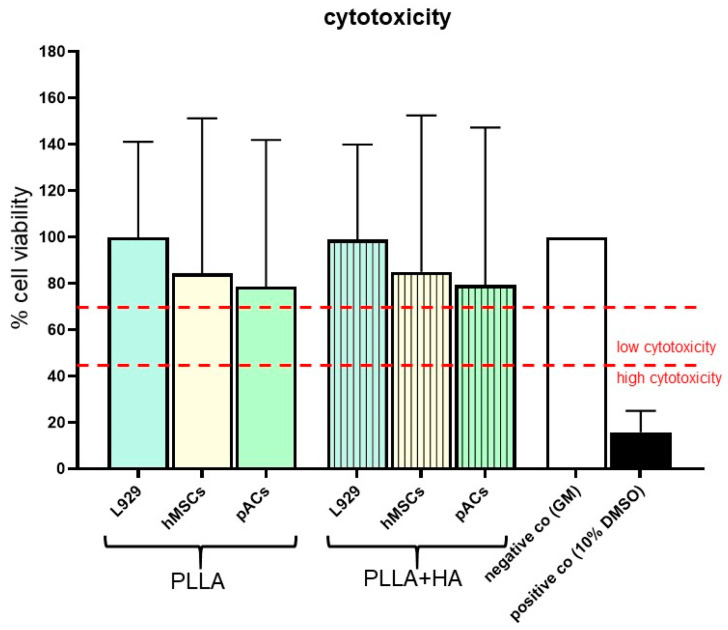
Cytotoxicity assay with bilayer PLLA scaffolds without/with HA supplementation. L929 fibroblasts, human mesenchymal stem cells (hMSCs) and porcine articular chondrocytes (pACs) precultured for 24 h in 96 well plates were exposed for 24 h to extracts of bilayer pure PLLA and PLLA + HA scaffolds (extraction time 48 h). Cytotoxicity was determined using MTS assay. co: control, DMSO: dimethyl sulfoxide, GM: growth medium, HA: hydroxyapatite, PLLA: poly-L-lactic acid. Mean values and standard deviations are shown. Statistical analysis was performed with One-way ANOVA and Tukey’s multiple comparison test. n = 5. The red lines mark different levels (low and high) of cytotoxicity.

Cytotoxicity of biphasic pure PLLA and PLLA + HA scaffolds was tested. No cytotoxicity could be shown for the extracts of scaffolds without or with HA using the cell line L929, which is generally recommended for cytotoxicity testing. Compared to the growth of the L929 exposed to both extracts, a slight suppression of the vitality of pACs and hMSCs was detectable, which did not exceed the threshold for cytotoxicity at 70% (Figure 3, upper red line).

### 3.3. hMSC Characterization

hMSCs used in the present study displayed the typical mesenchymal marker expression profile (positive: CD29, CD44, CD73, CD90, vimentin and negative: CD34, CD45) (Supplementary Figure S2). hMSCs exposed to adipogenic, osteogenic and chondrogenic induction media underwent multilineage differentiation confirmed by the formation of oil-red-positive pre-adipocytes as early as after 7 days. First, alizarin red positive pre-osteoblasts could be shown after 14 days, and an intensive calcium deposition indicated by alizarin red staining was evident after 28 days (Supplementary Figure S3). Chondrogenically induced cells that expressed cartilage proteoglycans were demonstrated after 21 days (Supplementary Figure S3).

### 3.4. Cell Survival and Colonization of the Scaffolds Supplemented with HA or Not

**Figure 4:**
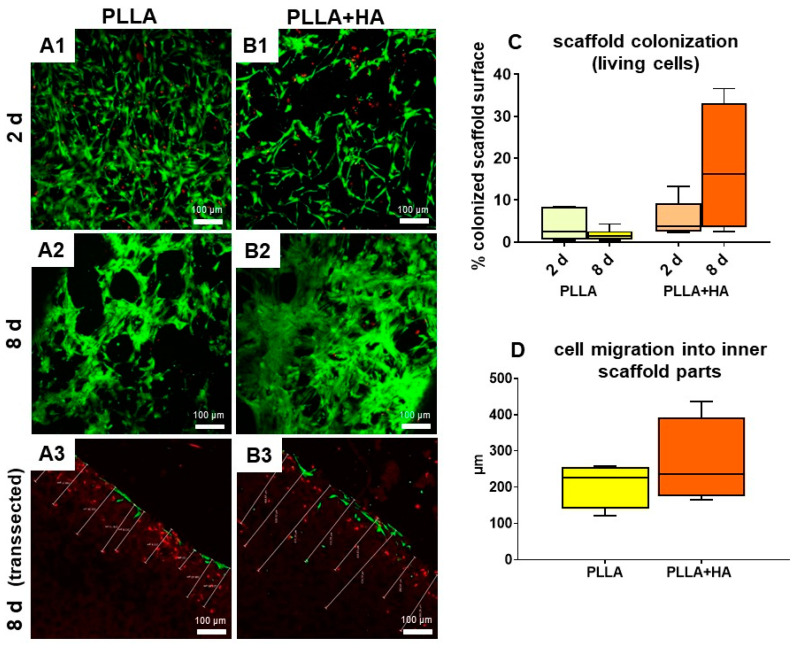
Adherence, survival, colonized surface area on unilayer PLLA scaffolds (100 µm pore size) without/with HA supplementation seeded with undifferentiated hMSCs after 2 and 8 days of statical culture. (**A1**–**A3**): PLLA, (**B1**–**B3**): PLLA + HA, (**A1**,**B1**): 2 days, (**A2**,**A3**,**B2**,**B3**): 8 days. (**A3**,**B3**): A transection through the middle of the scaffold is depicted. The scaffold surface seeded with hMSCs is shown on the upper side. (**C**): Scaffold surface area colonized with vital cells. (**D**): Cell migration into inner scaffold parts after one week. HA: hydroxyapatite, PLLA: poly-L-lactic acid. Red: dead cells, green: living cells. Scale bar: 100 µm.

Live/dead staining indicated that the majority of undifferentiated hMSCs survived on all scaffold variants tested for the whole observation time period. MSCs adhered to the surface of the scaffold but also filled pores or spanned their cell bodies through the pores attaching to their walls as shown by confocal laser scanning microscopy (Figure 4). Despite most cells exhibited an elongated shape, some rounded hMSCs were also detectable. An increase in cell density could be observed after 8 compared to 2 days of culturing hMSCs on the unilayer scaffolds. To prove this, scaffold areas colonized with vital cells were compared after 2 and 8 days for both scaffold variants (Figure 4). An increase in colonized scaffold areas could be detected after 8 days compared to 2 days for PLLA + HA (not significant), but not for pure PLLA.

In addition, the cell immigration into the inner regions of the scaffold was measured after 2 days using cross-sections of statical cultured unilayer scaffolds seeded with hMSCs using the live–dead assay (Figure 4). Furthermore, hMSCs penetrated in a mean of 237 ± 43 µm into the porous polymeric structure of all scaffolds. It has to be considered that some visible cells died due to cutting the scaffold into halves (red cells).

### 3.5. Osteogenic Differentiation

Osteogenically induced hMSCs were cultured for up to 21 days on both variants of unilayer scaffolds and showed mostly living cells (Supplementary Figure S3).

**Figure 5:**
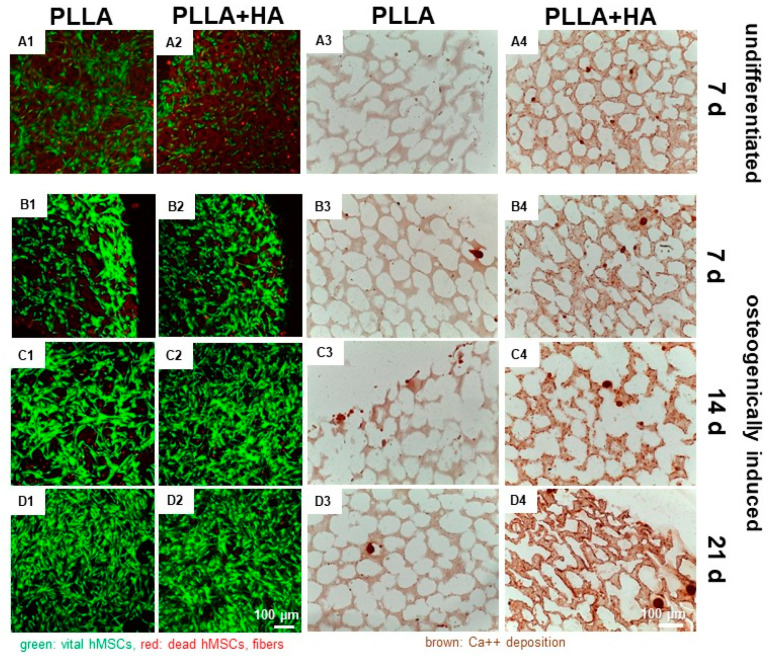
Adherence, survival and Ca^++^ deposition of non-induced and osteogenically induced hMSCs on unilayer PLLA scaffolds (**A1**–**D1**,**A3**–**D3**) or those supplemented with HA (**A2**–**D2**,**A4**–**D4**) after 7 (**A1**–**B4**), 14 (**C1**–**C4**) and 21 (**D1**–**D4**) days. **A1**–**B4**: Non-induced undifferentiated control. Red: dead cells, green: living cells. Ca^++^ deposition is visualized by alizarin red staining of non-induced or osteogenically induced hMSCs on unilayer PLLA scaffolds (**A3**–**D3**) or those supplemented with HA (**A4**–**D4**). Scale bars: 100 µm.

HE staining visualized mostly elongated cells but also some rounded cells. A denser layer of cells communicating with each other via cell extensions covered the surface of the scaffolds. However, MSCs could also be shown in the inner parts of the scaffolds (Figure 5).

Calcium deposition could be shown in scaffolds with HA supplementation and those seeded with osteogenically differentiated hMSCs, but also in the same scaffolds with undifferentiated hMSCs. PLLA without HA, seeded with undifferentiated cells, showed no Ca^++^ deposits. In contrast, when pure PLLA scaffolds were seeded with osteogenically differentiating hMSCs, Ca^++^ deposition was low, focally distributed and remained mostly cell-associated (Figure 5).

The hMSCs produced a fibrous ECM, which surrounded the cells and covered the scaffold surface. It was immunoreactive for collagen type 1, the most important ECM protein in bone, irrespective of whether the cells were osteogenically induced or not. Collagen type 10 is a marker of hypertrophic chondrocytes. It could be shown in all samples, when MSCs were osteogenically induced or even in undifferentiated cells cultured on HA-supplemented scaffolds (Supplementary Figure S4).

### 3.6. Cell Survival in Bilayer Scaffolds as Shown by Life–Death Assay

**Figure 6:**
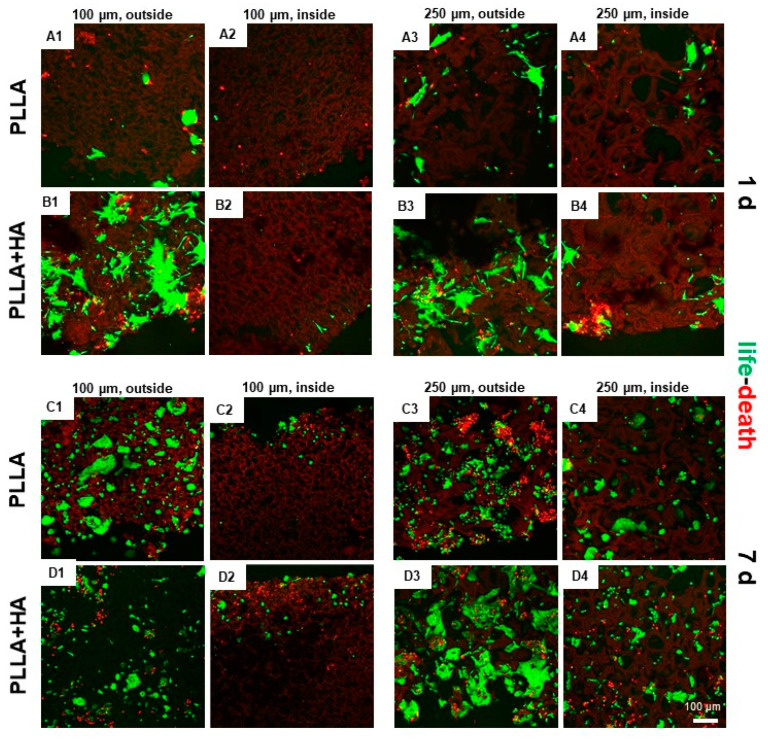
Cell survival on bilayer PLLA scaffolds without/with HA supplementation and seeded with undifferentiated hMSCs/pAC cocultures after cultured dynamically for 1 (**A1**–**B4**) and 7 (**C1**–**D4**) days. (**A1**–**D1**,**A2**–**D2**): 100 µm pore size, (**A3**–**D3**,**A4**–**D4**): 250 µm pore size. PLLA: (**A1**–**A4**,**C1**–**C4**), PLLA + HA: (**B1**–**B4**,**D1**–**D4**). HA: hydroxyapatite, PLLA: poly-L-lactic acid. Life–death assay: red: dead cells, green: living cells. Scale bar: 100 µm (representative for **A1**–**D4**).

In the next step, scaffolds consisting of two layers with different pore sizes were seeded with both, hMSCs cultured on the layer with 250 µm pores and pACs seeded on the layer with 100 µm pores. As a control, similar scaffolds seeded on both layers with hMSCs were cultured in the same manner. However, cell attachment and growth were generally lesser in the scaffold parts with smaller pore sizes (Figure 6). Most cells attached to the outer surface, but several cells could also be observed in the inner scaffold regions (Figure 6). The majority of cells survived on the scaffolds.

To check the inner parts, the unfixed scaffolds were dissected with a blade before microscopical images were taken. By this procedure, some cells were killed, which stained red. The hMSCs growing on the scaffold variants showed mostly an elongated morphology with cell cluster formation. The pACs appeared both, as elongated and round cells.

### 3.7. Cell Content and Glycosaminoglycan Deposition in Bilayer Scaffolds

For both assays, the scaffolds were dissected with a blade in the layers with the small (100 µm) and large (250 µm) pore sizes and analyzed separately. The hMSCs were seeded on the scaffold parts with larger pore sizes, whereas the pACs were placed on the layer with the smaller pores.

**Figure 7:**
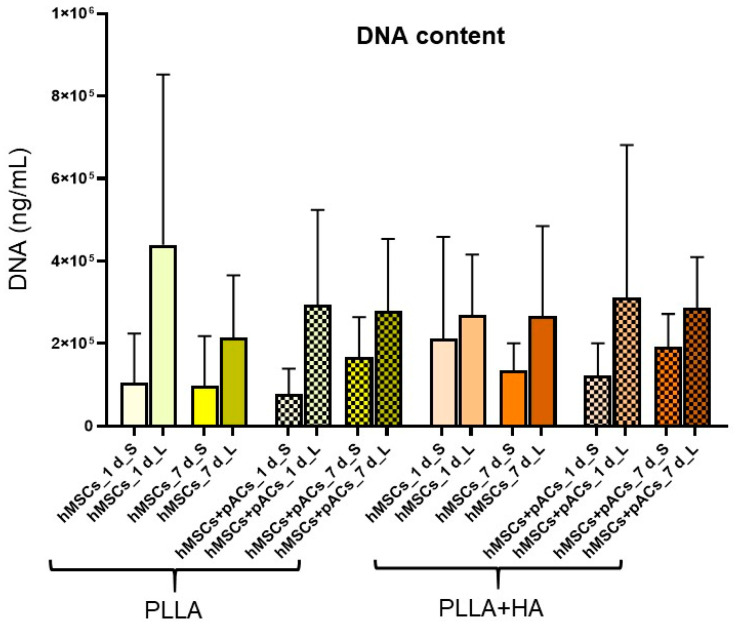
DNA content of hMSC/pAC cocultures and hMSC monocultures on bilayer PLLA scaffolds without/with HA supplementation after cultured dynamically for 1 and 7 days. HA: hydroxyapatite, hMSCs: human mesenchymal stem cells, L: larger pores, pAC: porcine articular chondrocytes, PLLA: poly-L-lactic acid, S: smaller pores. n = 6. Statistical analysis was performed with One-way ANOVA and Tukey’s multiple comparison test.

The DNA content was measured by the CyQuant assay (Figure 7). Despite the differences not being significant, the scaffold parts with the larger pore sizes (250 µm) showed a trend of higher DNA content compared to those with a 100 µm pore size. The comparison of the DNA content on the first day with that measured on the seventh day of scaffold culture revealed no significant differences (Figure 7).

**Figure 8:**
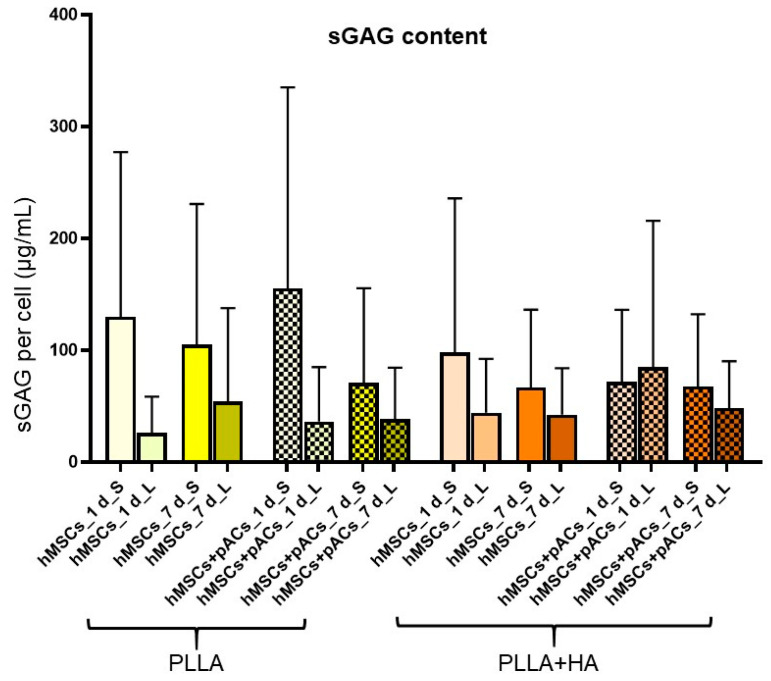
Sulfated glycosaminoglycan (sGAG) synthesis of hMSC/pAC cocultures and hMSC monocultures on bilayer PLLA scaffolds without/with HA supplementation after cultured dynamically for 1 and 7 days. HA: hydroxyapatite, hMSCs: human mesenchymal stem cells, L: larger pores, pAC: porcine articular chondrocytes, PLLA: poly-L-lactic acid, S: smaller pores. Statistical analysis was performed with One-way ANOVA and Tukey’s multiple comparison test.

In nearly all analyzed samples except for the PLLA scaffolds seeded with cocultures on the first day, the sGAG content was higher in the scaffold parts with the smaller pore size compared to the layer with larger pores (Figure 8). Nevertheless, the differences between the groups did not reach the significance level (Figure 8).

### 3.8. Cell Distribution and Cytoskeletal Architecture in hMSC/pAC Coculture on Bilayer Scaffolds as Shown by Immunolabeling

**Figure 9:**
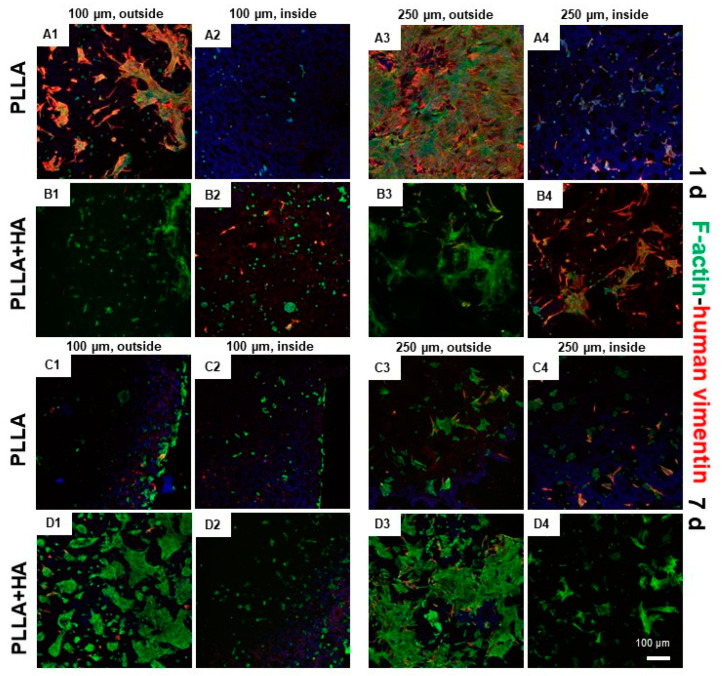
Cytoskeletal architecture of hMSC/pAC cocultures on bilayer PLLA scaffolds without/with HA supplementation after cultured dynamically for 1 and 7 days. (**A1**–**B4**): 1 day, (**C1**–**D4**): 7 days. (**A1**,**A2**,**B1**,**B2**,**C1**,**C2**,**D1**,**D2**): 100 µm pore size, (**A3**,**A4**,**B3**,**B4**,**C3**,**C4**,**D3**,**D4**): 250 µm pore size. PLLA: (**A1**–**A4**,**C1**–**C4**), PLLA + HA: (**B1**–**B4**,**D1**–**D4**). HA: hydroxyapatite, PLLA: poly-L-lactic acid. Red: human vimentin, green: F-actin, visualized with phalloidin Alexa488. Cell nuclei were stained with 4′,6-diamidino-2-phenylindole (DAPI). Scale bar: 100 µm (representative for **A1**–**D4**).

hMSCs were visualized by human-specific vimentin antibodies. To show all cells, this immunolabeling was combined with an F-actin stain, demonstrated by Phalloidin-Alexa488. Cell clusters of hMSCs could be detected on the scaffold surfaces or inside (mostly 250 µm pore size segments), which consisted mostly of elongated cells. All cells exhibited green F-actin fibers. The pACs were smaller than hMSCs and rounded. However, the hMSCs that were visualized by anti-human vimentin could not be exclusively detected on the 250 µm pore-sized scaffold segments but also on the layers with smaller pore sizes (Figure 9). One day after seeding, the inner parts of 100 µm pore-sized scaffold segments showed more exclusively green-stained pACs, and the inner parts of 250 µm pore-sized scaffold segments harbored more red-stained hMSCs. The vimentin staining of hMSCs decreased substantially at 7 days of culturing, especially in PLLA + HA scaffolds (Figure 9).

### 3.9. Gene Expression Profile in Bilayer Scaffolds

For the PCR analyses, the entire scaffolds were used for RNA extraction to ensure the acquisition of enough RNA. The samples were normalized to the expression in hMSC monolayers used as controls.

**Figure 10:**
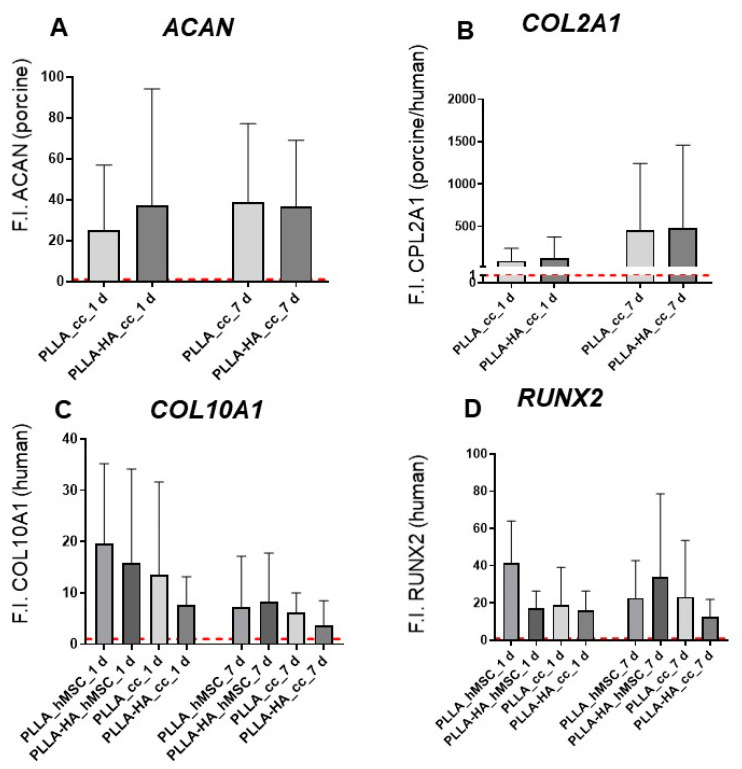
Gene expression analysis of hMSC/pAC cocultures and hMSC monocultures on bilayer PLLA scaffolds without/with HA supplementation after cultured dynamically for 1 and 7 days. (**A**): porcine *aggrecan (ACAN)*, (**B**): human and porcine *collagen type 2 alpha 1 chain (COL2A1)*, (**C**): human *collagen type 10 alpha1 chain (COL10A1)*, (**D**): human *runt-related protein 2 gene (RUNX2)*. HA: hydroxyapatite, PLLA: poly-L-actic acid. n = 3–5. The red line represents the respective gene expression level of the gene in a monolayer normalized to 1. Statistical analysis was performed with One-way ANOVA and Tukey’s multiple comparison test.

Cocultures of hMSCs and pACs showed stable *aggrecan* and *collagen type 2* expression at day 7 with no difference between scaffolds functionalized or not with HA. The hypertrophy marker *collagen type 10* and the early osteogenic marker runt-related protein *(RUNX)2* were expressed in all scaffold cultures more than twofold higher than in the hMSC monolayer. The hMSC monoculture on PLLA + HA scaffolds revealed an increase in *RUNX2* and *collagen type 10* between day 1 and 7; the cocultures on the same scaffold analyzed at day 7 expressed lesser *RUNX2* mRNA (both differences were not significant). (Figure 10).

## 4. Discussion

Due to their direct accessibility in the subchondral bone marrow and their capacity for osteogenic and chondrogenic differentiation, hMSCs are promising candidates for osteochondral defect repair. The pore size of the scaffold variants selected for the present study was in the typical range for scaffolds prepared using TIPS based on a ternary solution displaying evenly distributed HA particles inside the polymeric foam [9,22]. The topology of scaffold segments without and with HA did not show any differences. The uniform distribution of HA particles could be clearly visualized in the present study by microCT, suggesting that all cells in all scaffold areas might be exposed to HA. The HA particles demonstrated by microCT could also be seen in the alizarin red staining, the latter stains Ca^++^ deposits. The size of the HA particles chosen varied mostly extending between (0.05–1 µm), hence, including mostly nanoscaled particles with only some larger particles (10–100 µm). The selection of the HA concentration of 5% used in the present study was based on the results of previously published experiments, which showed that porosity decreased with increasing HA content and revealed that already 7% HA increased alkaline phosphatase activity in a pre-osteoblastic cell line [7,11].

A previous study reported cell retention, adherence and growth for a similar pore size range as selected in the present study (100 µm and 250 µm) for scaffold cultures of pACs and hMSCs [9]. It suggested that hMSCs might prefer larger pore sizes, whereas pACs might choose smaller pore sizes [9]. Therefore, for the scaffold segment in the bilayer scaffolds, which should mimic the osseous layer, the larger pore size range of 250 µm was selected, supplemented with HA to trigger the osteogenic differentiation of hMSCs. The upper segment containing 100 µm sized pores was designed for pACs to build up the future cartilage layer.

The high porosity of both layers allowed substantial cell penetration into the scaffolds even under static culture conditions, proving pore interconnectivity. This interconnectivity could also be seen histologically in scaffold sections. Accordingly, images could prove cell immigration into the inner scaffold parts. Cell seeding of bilayer scaffolds was challenging due to larger dimensions (6 mm height and 4 mm diameter), and a higher distance has to be permeated by nutrients by diffusion and cell migration. It is well known that there is a diffusion limit of around 250 µm in 3D constructs [23]. Nevertheless, hMSCs and pACs could also be found in the inner parts of the scaffold. The larger pore size showed higher numbers of attached cells, possibly due to superior accessibility and higher flow rates through the layers, which might facilitate cell nutrition.

After seeding and initial cell attachment, bilayer constructs were dynamically cultured. Dynamic culturing was superior compared with static approaches, as previously shown by others [7]. The rotation during dynamic culture applies shear forces, which might stimulate the hMSC differentiation.

The staining of the scaffolds seeded with hMSC/pAC cocultures with an antibody exclusively labeling human-derived cells (anti-human specific vimentin) visualized hMSCs but not pACs, whereas both, hMSCs and pACs exhibited green-stained F-actin stress fibers. hMSCs and pACs could be localized both, at the outer and inner surfaces of the scaffolds. The actin cytoskeleton is involved in cell migration. The co-staining revealed that after 1 day of culturing, the inner parts of the 250 µm pore-size scaffold area contained, as expected, mainly hMSCs, whereas the outer borders also showed some mixtures of hMSCs and pACs. In the inner parts of the scaffold, the segments with 100 µm pores contained, as estimated, pACs as the dominating cell population. This indicates that coculture seeding was successful. Nevertheless, after one week of culturing, the human-specific vimentin staining of the hMSCs decreased, but the larger cell size and fibroblast-like morphology of hMSCs still allowed their distinction from the pACs. The reason for the decrease in visible vimentin fiber networks in hMSCs remains unclear but could be influenced by the longer time of hMSC transition from monolayer to 3D scaffold culture associated with cytoskeletal reorganization. Nevertheless, vimentin is known to be involved in chondrocyte cellular stiffness and mechanoresponsiveness [24]. Most of the cells survived on the scaffolds, irrespective of supplementation with 5% HA. The staining reflected the presence of only low numbers of dead cells. To check the cell content of inner scaffold parts, scaffolds had to be sectioned, which led to some visible cell injury at the cutting edges and, accordingly, more dead cells. Scaffold functionalization with 5% HA stimulated hMSC spreading since the mean colonized area on the unilayer scaffolds increased after 8 days compared to the first observation time point of 2 days, but only in the scaffolds supplemented with HA. This was generally higher in the HA-supplemented unilayer scaffolds than in those without supplementation. This observation is in agreement with a previous report that HA in gels might facilitate chondrocyte and MSC migration [25].

Bilayer scaffolds seeded with hMSCs alone or hMSC/pAC cocultures revealed the gene expression of ECM components, whereby no significant effect of HA supplementation could be shown compared to the pure PLLA scaffolds. In this study, bilayer scaffold cultures were kept in a non-inductive growth medium to exclusively show the effects of HA supplementation and not influence the chondrocyte phenotype of the pACs in cocultures by exogenic additives or growth factors.

Stable cartilage-specific *collagen type 2* and *aggrecan* gene expression indicated the chondrogenic phenotype in hMSC/pAC cocultures on the bilayer PLLA scaffolds. The gene activity of the hypertrophic marker *collagen type 10* and the early osteogenic marker *RUNX2* could also be detected in all cultures, suggesting the start of osteogenic differentiation. However, the observation time of 7 days is too short for stable osteogenic differentiation. In contrast to an in vivo scenario within an osteochondral defect, both scaffold layers colonized either with pACs or hMSCs were kept under similar growth conditions using the same growth medium, and for this reason, a short culturing time was preferred. The aim was to focus on the very important initial steps of cell–bilayer scaffold interaction. To distinguish the cell populations on each layer of the bilayer scaffolds, human-derived MSCs and ACs of porcine origin were combined; hence, interspecies immunological interactions cannot be excluded. A limitation of the gene expression analysis was that both scaffold layers with different pore sizes were not separately studied to obtain enough RNA for analyses.

An exemplary long-term experiment with hMSCs cultured on unilayer PLLA and PLLA + HA scaffold variants demonstrated the osteogenic induction of hMSCs after the end of the observation time (21 days): an enhanced Ca^++^ deposition in scaffolds supplemented with HA, which were kept in osteogenic culture medium, became evident.

In agreement with this observation, significant differences in the alkaline phosphatase activity of the pre-osteoblastic MC-3T3-E1 cell line either seeded on PLLA unilayer scaffolds without or with HA could be found later at 21 days of culture [11]. In contrast to the present study, in the cited study, the unilayer scaffolds were additionally coated with collagen for better cell adhesion [11].

## 5. Conclusions

The present study proved the feasibility of preparing an osteochondral bilayer scaffold using TIPS. In addition, a successful bilayer seeding strategy was established and used to perform a coculture of osteochondral cells on these highly porous PLLA scaffolds supplemented with HA. Future experiments should try to optimize the seeding and dynamical culturing procedure, e.g., by using a bioreactor [7].

hMSCs and pACs cocultures survived on bilayer scaffolds and also colonized inner parts of the bilayer scaffolds, indicating the functional interconnectivity of the scaffold pores. In regard to cell retention, the layer with larger pore sizes was superior, supported by a higher proportion of adhering cells and higher mean DNA concentrations. Cells reflected osteogenic and cartilage-specific marker expressions as a promising step towards osteochondral constructs.

## Figures and Tables

**Table 1:** Antibodies and stains used to depict proteins of hMSCs and pACs.

Target	Primary Antibody	Dilution	Secondary Antibody	Dilution
CD29 (β1-integrin)	mouse anti-human, Merck KGaA, Darmstadt, Germany	1:50	donkey anti-mouse cy-3, Invitrogen, Carlsbad, CA, USA	1:200
CD34	mouse anti-human, BD Pharmingen, San Diego, CA, USA, APC labeled	1:50	donkey anti-mouse cy-3, Invitrogen	1:200
CD44	mouse anti-human, Cell Signalling Technology, Danvers, MA, USA	1:70	donkey anti-mouse A488, Invitrogen	1:200
CD45	mouse anti-human, DAKO, Cytomation, Hamburg, Germany	1:70	donkey anti-mouse cy-3, Invitrogen	1:200
CD90	mouse anti-human, BD Pharmingen, FITC labeled	1:70	donkey anti-mouse A488, Invitrogen	1:200
CD105	mouse anti-human, Miltenyi, Bergisch Gladbach, Germany, PE-labeled	1:50	donkey anti-mouse cy-3, Invitrogen	1:200
collagen type 1	goat anti-human, Abcam, Cambridge, UK	1:50	donkey-anti-goat cy-3, Invitrogen, Carlsbad, CA, USA	1:200
collagen type 10	mouse-anti human, Abcam, Cambridge, UK	1:50	donkey anti-mouse cy-3, Invitrogen	1:200
4′,6′-diamidino-2-phenylindol (DAPI)	Roche, Mannheim, Germany	1:100	-	1:200
phalloidin-A488	Santa Cruz Biotechnologies Inc., Dallas, TX, USA	1:100	-	1:200
vimentin	mouse anti-human, Dako Cytomation, Hamburg, Germany	1:50	donkey anti-mouse cy-3, Invitrogen	1:200
anti-human-specific vimentin	rabbit anti-human, Zytomed systems, Berlin, Germany	1:50	donkey anti-rabbit A555, Invitrogen	1:200

A488: AlexaFluor488, A555: AlexaFluor 555, CD: cluster of differentiation, cy-3: cyanine 3.

**Table 2:** Primers used for realtime detection PCR experiments with seeded bilayer scaffolds.

Gene Symbol	Species	Gene Name	Efficacy	Amplicon Length (Base Pairs)	Assay ID
*ACAN*	*Sus scrofa*	*aggrecan*	1.69	60	Ss03374823_m1
*ACTB*	*Homo sapiens*	*β-actin*	1.89	171	Hs99999903_m1
*ACTB*	*Sus scrofa*	*β-actin*	1.71	77	Ss03376081_u1
*Col2A1*	*Homo sapiens*	*collagen type 2*	2.06 (1.9 *)	124	Hs00264051_m1
*RUNX2*	*Homo sapiens*	*Runt-related protein*	1.94	116	Hs00231692_m1
*COL10A1*	*Homo sapiens*	*collagen type 10 alpha 1*	2.15	76	Hs00166657_m1

* efficacy determined for cDNA of *sus scrofa*.

## Data Availability

Data can be provided upon request.

## Supplementary Materials

The following supporting information can be downloaded at: https://www.mdpi.com/article/10.3390/polym16030331/s1. Figure S1: Hydroxyapatite (HA) particle dimension analysis and scanning electron microscopic visualization. The percentage of HA particles with a specific dimension was analyzed and confirmed via SEM analysis; Figure S2: Characterization of undifferentiated hMSC surface marker expression profile. A: CD29 (red), B: CD34 (red), C: CD44 (green), collagen type 1 (red), D: CD45 (red), E: CD73 (red), F: CD90 (green), vimentin (red), G: negative control. Cell nuclei are counterstained in blue. Scale bars: 50 µm; Figure S3: Multilineage potential of bone marrow-derived MSCs. A, C: Oil red staining of adipogenically induced hMSCs after 7 days and B, D: Alizarin red staining of hMSCs cultured for 28 days on cover slides. E: Alcian blue stain of chondrogenically induced (21 days) hMSC pellets. Scale bars: A, C: 50 µm, B, D: 100 µm, E: 200 µm; Figure S4: Expression of collagen types 1 and 10 of non-induced and osteogenically induced hMSCs on unilayer PLLA scaffolds. Collagen type 1 is depicted in red, collagen type 10 in green. 4′,6-diamidino-2-phenylindole (DAPI) was used to counterstain cell nuclei. Scale bars: 100 µm.
