# Supplementary material for: The Phenotype of Mesenchymal Stromal Cell and Articular Chondrocyte Cocultures on Highly Porous Bilayer Poly-L-Lactic Acid Scaffolds Produced by Thermally Induced Phase Separation and Supplemented with Hydroxyapatite
Source: Polymers (Basel). 2024 Jan 25;16(3):331. doi: 10.3390/polym16030331 (PMC10857361; doi:10.3390/polym16030331)
Supplement: Supplementary file 1 [file polymers-16-00331-s001.zip › polymers-2760716-supplementary.pdf]

Supplementary Materials

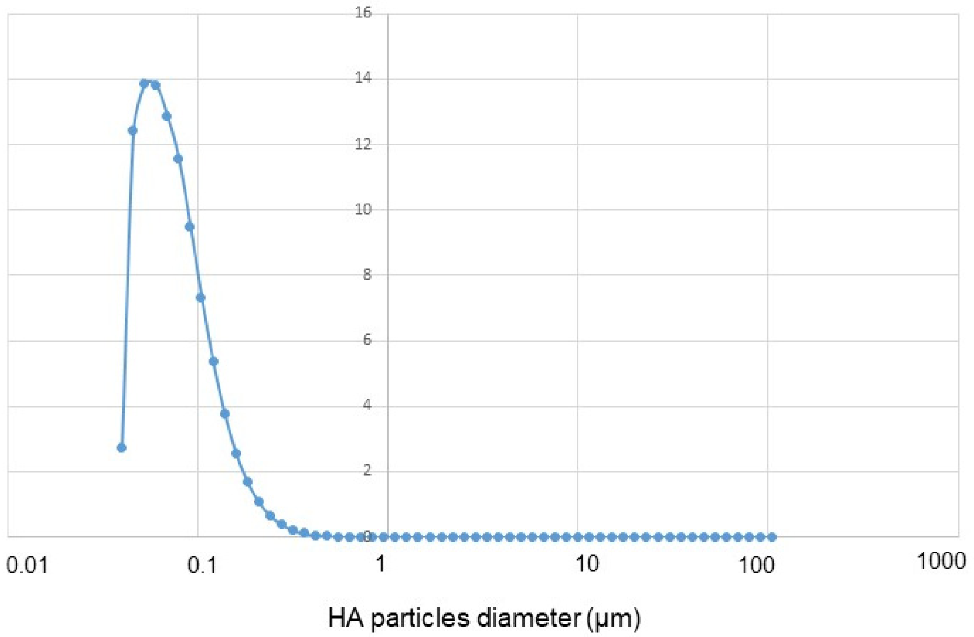

A

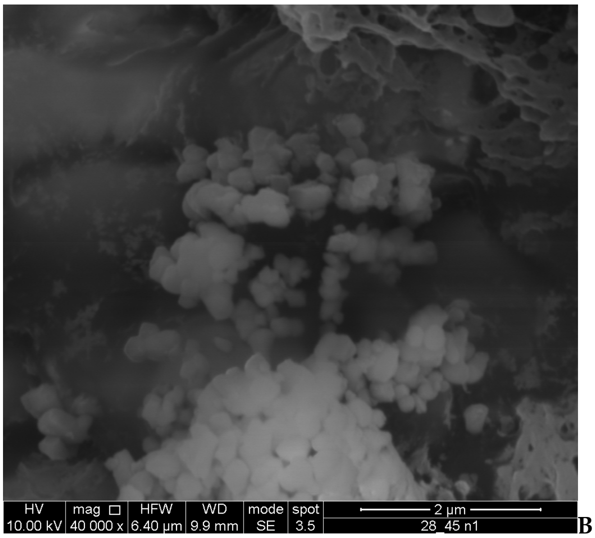

B

Figure S1. Hydroxyapatite (HA) particle dimension analysis and scanning electron microscopic visualization. The percentage of HA particles with a specific dimension was analyzed and confirmed via SEM analysis.

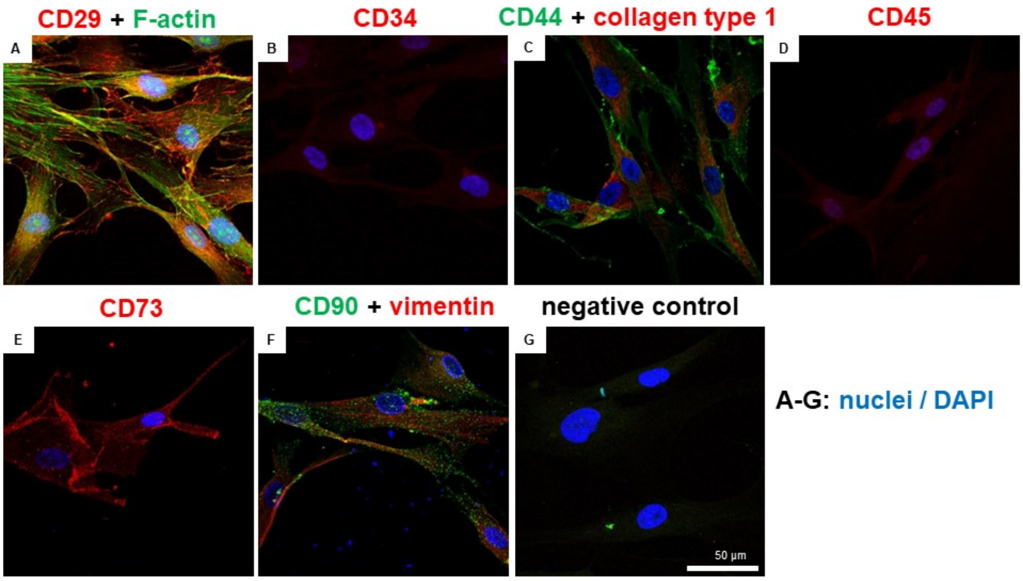

Figure S2. Characterization of undifferentiated hMSC surface marker expression profile. A: CD29 (red), **B:** CD34 (red), **C:** CD44 (green), collagen type 1 (red), **D:** CD45 (red), **E:** CD73 (red), **F:** CD90 (green), vimentin (red), **G:** negative control. Cell nuclei are counterstained in blue. Scale bars: 50 µm.

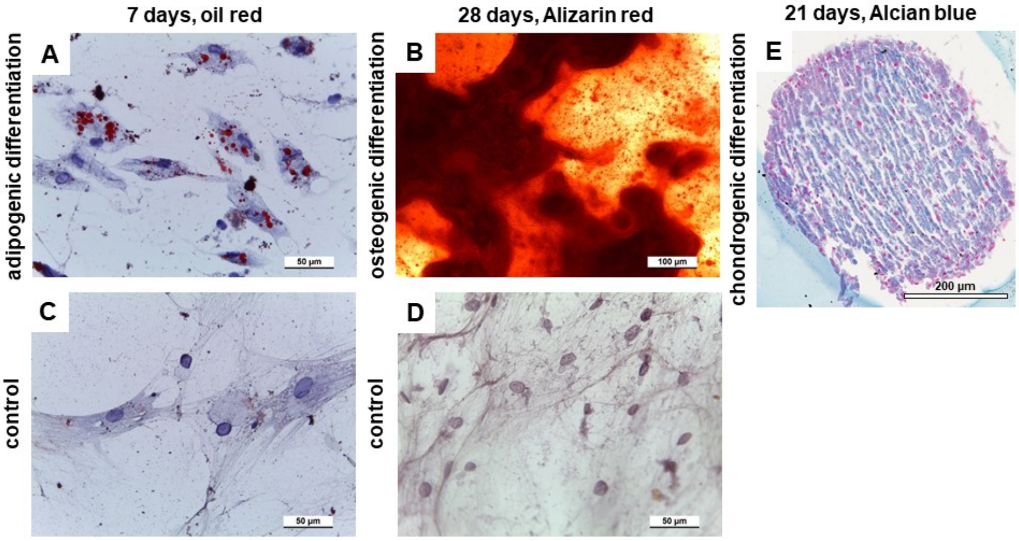

Figure S3. Multilineage potential of bone-marrow-derived MSCs. A, C: Oil red staining of adipogenically induced hMSCs after 7 days and **B, D:** Alizarin red staining of hMSCs cultured for 28 days on cover slides. **E:** Alcian blue stain of chondrogenically induced (21 days) hMSC pellets. Scale bars: **A, C:** 50 µm, **B, D:** 100 µm, **E:** 200 µm.

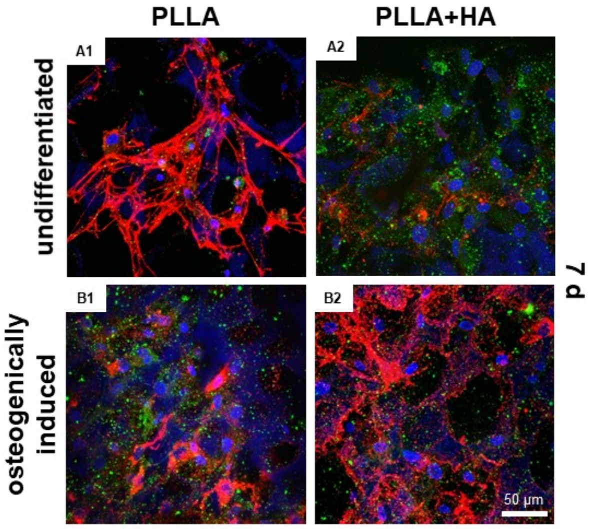

Figure S4. Expression of collagen types 1 and 10 of non-induced and osteogenically induced hMSCs on unilayer PLLA scaffolds. Collagen type 1 is depicted in red, collagen type 10 in green. 4',6-diamidino-2-phenylindole (DAPI) was used to counterstain cell nuclei. Scale bars: 100 µm.
